# Depression and Exercise in Older Adults: Exercise Looks after You Program, User Profile

**DOI:** 10.3390/healthcare10020181

**Published:** 2022-01-18

**Authors:** Carmen Galán-Arroyo, Damián Pereira-Payo, Miguel Ángel Hernández-Mocholí, Eugenio Merellano-Navarro, Jorge Pérez-Gómez, Jorge Rojo-Ramos, Jose Carmelo Adsuar

**Affiliations:** 1 Promoting a Healthy Society Research Group (PHeSO), Faculty of Sport Sciences, University of Extremadura, 10003 Cáceres, Spain; magaar04@alumnos.unex.es (C.G.-A.); jadssal@unex.es (J.C.A.); 2 Health Economy Motricity and Education (HEME), Faculty of Sport Science, University of Extremadura, 10003 Cáceres, Spain; dpereirab@alumnos.unex.es (D.P.-P.); jorgepg100@unex.es (J.P.-G.); 3 Physical Activity and Quality of Life Research Group (AFYCAV), Faculty of Sport Science, University of Extremadura, 10003 Cáceres, Spain; mhmocholi@unex.es; 4 Grupo de Investigación EFISAL, Universidad Autónoma de Chile, Talca 3460000, Chile; emerellano@gmail.com; 5 Social Impact and Innovation in Health (InHEALTH), University of Extremadura, 10003 Cáceres, Spain

**Keywords:** depression, elderly, health-enhancing physical activity (HEPA), prescription exercise, health-related quality of life (HRQoL), Exercise Looks After You (ELAY)

## Abstract

Introduction: Depression is a challenge for public health policies, as it is the number one leading cause of disability in the world. In order to combat and prevent it, different social and health interventions are being developed to promote health through physical activity. Objective: Analyze and describe the user profile of the patients with depression from the Exercise Looks After You program, which is a physical activity program that works on improving public health and has an essential role preventing chronic diseases and improving the quality of life of the elderly in Extremadura. Design: Cross-sectional study. Participants: total sample of 1972 users (96.4% women, 3.6% men), of whom 724 (94.6% women, 5.4% men) suffer from depression. Results: It was observed that the dominant user profile of the patients with depression within the program is female, 71 years old, physically active, overweight, married, with low educational level, non-smoker, no alcohol consumption and below average physical fitness and health-related quality of life, which translates into a high incidence of primary care, nursing and prescription visits. Conclusions: This study presents the user profile of depressive versus non-depressive participants of the Exercise Looks After You physical activity program. This data could be meaningful in order to improve and optimize public health programs and resources.

## 1. Introduction

Depression is a mental disorder that incapacitates millions of people worldwide [1,2]. It is characterized by persistent apathy and continuous sadness, which alters the quality of sleep and appetite of the patients, causing fatigue, lack of concentration and low self-esteem [3], reducing psychic functions and thus decreasing the quality of life of people who suffer from it [4,5].

Worldwide, depression represents the fourth leading cause of mortality and the leading cause of disability in terms of loss of healthy life years [6]. In Europe, it is one of the biggest public health concerns [7], in terms of prevalence, disease burden and disability [8,9]. In Spain, it is the mental disorder with the highest prevalence [10]. Nonetheless, it is currently underdiagnosed [11]. The lack of response and the persistence of residual symptoms in treated patients who do not fully recover [12] have turned depression into a real public health problem in our country [3].

In the older population the scenario is even worse, as between 6 and 10% of adult seniors suffer from depression [13]. With aging, this mental disorder causes changes at the psychological level and decreases emotional well-being [14]. With depression, physical activity levels also decrease and health worsens [15]. Furthermore, sedentary behavior (SB) is considered a risk factor for depression [16], increasing the likelihood of metabolic disorders, such as type 2 diabetes and cardiovascular diseases [17]. This mental disease has been associated with increased disability and mortality [18], leading to a decrease in life expectancy of 8 to 10 years [19].

Recent studies have demonstrated the usefulness of physical activity (PA) as a pre ventive strategy and as a therapy (supplemental or alternative) for mental illness [20,21]. Decreasing physical inactivity rates should be a main objective [22]. In fact, there is a correlation between low levels of PA and the presence of anxiety and depression symptoms in older people [23]. Therefore, one of the key purposes in public health is to promote an active lifestyle through health-enhancing physical activity (HEPA), since PA is presented as an effective preventive tool against depression [24].

Regarding the treatment of depression, pharmacological therapy is the most used method, which on many occasions is insufficient, due to the patient’s resistance to antidepressant drugs [25] since a high percentage of the patients present previous anxiety disorder episodes [26] and also due to the side effects of the drugs [27], which cause alterations in the physical, social and cognitive functioning of the patients. This entails a high cost for health systems [28], now more than ever. Depression has been addressed using alternative types of therapy such as PA and physical exercise (PE) [29].

Several studies, reviews and meta-analyses ensure that PE is indicated to reduce and treat mental health disorders [30] and for reducing stress and anxiety levels [31]. Moreover, PE has a lot of influence on the well-being of an individual [32], thus it could be helpful to decrease medication intake and improve the disease overall [33]. Therefore, PA and PE are recommended as the main therapy for the clinical treatment of subclinical, mild and moderate depressive symptoms [29,34]; and for severe depression, they are recommended as a parallel therapy [35], and as a preventive and/or treatment strategy [36,37], their efficacy has been demonstrated [38].

In the last decade, a large number of primary care-based interventions have been developed [39]. They are being designed based on the use of ERS (exercise referral schemes) programs that help inactive adults with chronic diseases to become physically active [40]. In relation to depression, almost all studies on PA have consisted of randomized control trials (RCTs) and have focused on structured PE; they have reported improvements in perceived health and physical and mental improvement in day-to-day life [41]. Unfortunately, there is little scientific evidence regarding the information of the profile of the patient with depression who participates in a physical exercise public program. Describing the user profile of the patients with depression that take part in public physical exercise programs could be relevant in order to know whether it would be possible to optimize the program, improve assistance, know the different comorbid diseases that are associated with depression and know if it would be necessary to update the portfolio of public health services and orientate the objectives and adapt contents according to the profile, since the exercise predilection is known: men prefer strength work and women prefer aerobic work and stretching [42]. This information could help to increase male participation in the programs which is significantly lower than female participation, (94.6% women, 5.4% men FTE Data 2019); but above all, describing the user profile of the patients with depression would allow us to optimize public health guidelines and recommendations.

Considering the effectiveness of these programs, Exercise Looks After You (ELAY, 2006), a HEPA public walk-based health program with the purpose of promoting PA and healthy lifestyles in older-age individuals, was created in Extremadura, Spain. This program is an example of the comprehensive and integrated approach to health when it is well executed [43]). This PA intervention which is linked to the public health system combines multidisciplinary work between health and sports science professionals. The program oriented to elderly people aims to contribute, among other objectives, to prevent dependence, favor personal autonomy and improve the quality of life of elderly people with health problems through its socio-health and specialized interventions, specifically to provide the target population with a service based on physical exercise (3 days per week) to improve or preserve the different components involved in healthy physical condition. Its design responds to the following recommendations put forward by the World Health Organization: 150 min of weekly moderate aerobic physical activity or 75 min of vigorous physical activity, strengthening activities of the main muscle groups at least twice a week and balance development and fall prevention activities at least 2 times per week. Results have shown that the practice of physical exercise reduces medical consultations by 29% [44,45] and significantly improves health-related quality of life (HRQoL) measured through the EQ-5D-3L test, especially in the dimensions of pain–discomfort and anxiety–depression [46]. The profile or baseline data of the overall participants of the program has also been reported [47,48] and a considerable reduction (68%) from mild, moderate or severe depression to no depression has been demonstrated for the participant group [49].

Given the importance of the use of this service in public health, it is necessary to analyze the profile of the ELAY user with depression, in order to improve the quality of the program and to develop effective strategies to attract older people with depression with the aim of improving health and community policies in Extremadura.

## 2. Materials and Methods

Methodology used in the study is based on the analysis of the data obtained from the evaluations collected during 2019 by the technicians of the ELAY program.

### 2.1. Design

This is a cross-sectional study which describes the values of the dimensions studied that allow us to know the user profile of the patients referred from primary care with a diagnosis of depression to the Extremadura public health program ELAY.

### 2.2. Sample

A total of 1972 users participated in the program, of whom 724 had depression (according to the Geriatric Depression Scale (GDS), a screening instrument to detect depression in the elderly [47]), 685 women and 39 men.

The data of the ELAY participants was treated with complete confidentiality, in compliance with the data protection law. The participants received the benefits/risks of participation in the study and subsequently voluntarily signed the informed consent form, in compliance with the Declaration of Helsinki. The study was approved by the Biomedical Ethics Committee of the University of Extremadura (117/2021).

Sample inclusion criteria:

The population included in the study comprises: people over 59 years of age who are users of the ELAY PA program. To be a user they must meet the following requirements: male or female resident in Extremadura, referrals from primary care and without medical contraindication that would prevent completion of CFS test battery.

### 2.3. Measures and Instruments

The instrument used to measure depression was the Yesavage Scale for Geriatric Depression in Spanish version (GDS-VE) [50]. This scale quantifies depressive symptoms and is widely used in older adults. It only explores cognitive symptoms of a major depressive episode, with a dichotomous response pattern to facilitate completion. It is a validated instrument, easy to answer and created specifically for the elderly population. A score of 0 to 4 means no depression, 5 to 8 means mild depression, 9–11 means moderate depression and 12 to 15 means severe depression [51].

Sociodemographic variables (age, sex, marital status, educational level, weekly physical activity level and habits related to alcohol and tobacco consumption) were obtained through a specific ELAY questionnaire.

The Spanish version of the EQ-5D-3L (EuroQol5Dimensions-3Levels) questionnaire [52] was used to measure HRQoL. It consists of six sections. The first five summarize HRQoL in five dimensions (mobility, self-care, activities of daily living, pain/discomfort and anxiety/depression), each containing three response levels (no problems, some problems or many problems) [53].

For bi-manual grip strength, measurements were performed on both hands using a grip dynamometer, (TKK 5401). Two measurements were made with each hand and the maximum value of each hand was added [54]. The relative reliability of this test was reported in a Spanish adult population with an ICC of 0.99 [54].

Shoulder flexibility was assessed through the “back scratch” test [55] where the distance between the third finger of both hands was recorded, considering a positive value if the hands overlap and a negative value if the hands do not reach each other. For this test a reliability of ICC = 0.96 was reported [55].

For trunk and lower limb flexibility, the adapted seated sit-and-reach test [56] was performed, in which after two trials (one with each leg) the best score (right or left) was recorded. For this test, relative reliability was reported with an ICC = 0.95 [56].

The functional reach test was performed to measure static balance [57], where the maximum distance achievable with the arms extended, flexing the trunk without moving the feet off the ground, was recorded.

Agility was measured using the 3-m version of the “Time, up & go” test [58], where the participant started in a sitting position, then they had to stand up, go around a cone that was placed 3 m away and return to sitting down. Performance in the test is measured with the time in seconds from the moment the start signal is given until the person adopts the sitting position with the back fully supported on the back of the chair. The best result obtained from two attempts was recorded. Good reliability indices have been reported in both tests, ICC = 0.81 in the functional reach [57] and ICC = 0.98 in the “Time, up & go” [58].

The “6-min walk” test was performed to assess the cardio-respiratory endurance of the participants. It consists of recording the maximum distance that a participant can walk for 6 min in a 20-m corridor [55]. Only one trial was performed for this test. The reliability reported for it is ICC = 0.94 [56].

Additionally, body composition measurements were taken. Weight and height were recorded (Seca 780; Seca Ltd., Birmingham, UK). Furthermore, the recommendations established by the Council of Europe [59] for the calculation of body mass index (BMI) were followed.

### 2.4. Statistical Analysis

The analysis of the data collected was performed with the Statistical Package for Social Sciences (SPSS) version 23.0 for MAC (IBM Corporation, Armonk, NY, USA).

First, the Kolmogorov Smirnov test was performed to determine whether the data followed a normal distribution; since this assumption was not met, it was decided to use nonparametric tests. Statistical significance was established at *p* < 0.05.

Pearson’s chi-square test was used to analyze the differences in the variables (sex, body mass index, educational level, alcohol and tobacco consumption and level of physical activity) according to the group (persons with or without depression). Statistical significance was established at *p* < 0.05.

Mann–Whitney U test was used to analyze the relationships between the variables (Falls, Primary Care visits, Nurse_Visits, Specialist_Visits, Prescription_Visits, Hospital Days, Handgrip, Seat-and-Reach, Back Scratch, Functional Reach, Stand Up, Time Up and Go, 6 min Walk and EQ-5D as a function of the group (people with or without depression).

## 3. Results

Table 1 shows the distribution of ELAY program users according to the depression variable (36.4% suffer from depression) and according to sex (94.6% women and 5.4% men).

Table 2 describes the sociodemographic characteristics of the users: age (71.6 years), BMI (overweight 45.7% and obese 42.1%), marital status (married 68.1%), educational level (no education 36.9% and primary education 49.3%), alcohol and tobacco consumption (non-drinking 83.3% and non-smoking 95.4%) and level of physical activity (>3 h/week 91.9%).

Table 3 shows the use of the Extremadura health service in terms of primary care visits. There is a significant difference in visits to the general practitioner, visits to the nurse, visits to the specialist and visits for prescriptions.

## 4. Discussion

The main finding of this study was the description of the user profile of the patients with depression from the ELAY program. To the best of our knowledge, it is the first article to do so. According to the applied criteria (ranked above 5 in the GDS questionnaire, according to Yesavayage [51]), 36.7% of the users suffer important depressive symptoms. This could be meaningful data, given that users are only tested if they get diagnosed with depression and/or have prescribed medication, which means that many users do not take this test. Further, in the elderly population depressive symptoms are difficult to diagnose because they are often confused with aging [60].

Regarding gender, we can highlight that there is a significantly higher percentage of women (94.6%) attending the HEPA program than men (5.4%), along the lines of the proactive physical exercise program in the elderly by Piñeiro et al. where participation was exclusively female [60].

Users have a mean age of 71.55 years. In relation to marital status, they are mostly married. There were no significant differences with non-depressives, as in the study by Perez-Souza et al. [49].

Generally, most of the patients have low educational levels: primary school or no education at all; this is in line with the Norwegian study [61], which stated that a high proportion of HEPA program users had low educational level and low income.

Regarding the level of physical activity, the participants were physically active more than three times per week, which makes them physically active people, according to the WHO recommendation criteria. Physical activity has been shown to have a protective effect against the emergence of depression regardless of age and geographical region [16,62].

Concerning body composition, the majority of the users were overweight (45.7%) or obese (42.1%), in line with the study conducted in the North West of England, UK, which showed a varied health profile characterized by high levels of obesity (27%), although in their case, low levels of PA (48.3% achieving 150 min of PA weekly) [63]. In the current study, only users who were already initiated in the program were registered; therefore, their mean level of physical activity is higher. Body composition could be a matter of concern regarding depressive disorder, as an association between increased body weight and the development of depressive disorder has been observed in some studies [64,65]. In adults and aged adults, obesity and overweight increased the risk of onset of depression [64]. The association between depression and having obesity or being overweight could be occasioned by poorer self-image and lower self-esteem levels of the participants with depressive disorder due to the weight stigma in today’s society [65,66,67]. These changes in body weight could be explained by some behavior typically repeated by the patients with depressive disorder, such as increased sleepiness and appetite, that have been found to have more prevalence for women with depression [62,65]. Regarding alcohol and tobacco consumption habits, there was no significant difference between users who have depression and those who do not. Some studies reported that the participants did not smoke and did not drink any alcohol [49]. In relation to medical care in the Extremadura health service, there is a statistically significant correlation in the variables: primary care visits, nursing visits and visits for prescriptions; where people with depression accounted for a higher medical expenditure, in line with the studies of the Mental Health and Wellbeing Report, 2016 that described the high cost of this disease for health services [64] and in line with Gusi’s cost-utility study [45].

Regarding physical fitness parameters, there is a significant difference *p* < 0.01 in the seat and reach test, stand up, time up and go and 6 min walk test. These results are similar to previous studies [67,68,69,70]. As some authors have shown poor physical performance is a predictor of future onset of depression in elderly adults [71,72,73], it seems that subjects with poorer performance in balance, gait speed and strength have greater probabilities of suffering depressive disorders in the future [72].

As for the quality of life assessed by the EQ-5D, it can be seen that there is a substantial improvement among people who have depression compared to those who do not have it. There are several studies that show improved quality of life in older people who do not have depression [74,75], although it is more difficult to find specific ones.

### Limitations of the Study

It is important to emphasize that this study has certain limitations that should be taken into consideration. On the one hand, we cannot take the sample size as representative, since we have a reduced number of participants with respect to the real number of people who suffer from depression and are not referred to the program due to lack of diagnosis.

On the other hand, the referral is carried out by the primary care team with a diagnosis of depression, but we do not know what type of depression they suffer from, since we cannot access this information and the GDS questionnaire serves to know which depressive syndromes they suffer from but does not diagnose.

Finally, it is important to emphasize that the number of people who are referred to the program is unknown. The investigators only knew the number of patients who accepted taking part in the program. Knowing the volume of patients referred to a HEPA program and, moreover, the characteristics of those referred are critical to begin to interpret the generalizability of the findings to the broader population [76].

As future lines of research, it would be interesting to be able to study variables such as the average expenditure of the patient with depression in terms of medication and services to know the cost effectiveness of this type of HEPA program and its impact on public health. In addition, it would be useful to know the motivation of people with depression who participate in the program and thus be able to offer a specific response according to their needs so that they do not drop out and to include healthy lifestyle habits oriented to socialization in order to be more explicit in the actions and/or protocols to be carried out. Finally, it would be interesting to promote a new concept of physical exercise program for health, which helps to dismantle the established roles and try to establish a community service where PE is really valued as a preventive and/or palliative tool in chronic diseases.

## 5. Conclusions

In conclusion, the user profile of the patient with depression who participates in a public health HEPA program (ELAY) in Extremadura is female, in general with a septuagenarian profile, married, with a low academic level of studies, physically active, does not smoke or consume any alcohol, is overweight and has below average physical fitness and health-related quality of life, which causes a high incidence in primary care visits, nursing and visits for prescriptions. These data lead us to look for exercise options oriented to men, promoting concrete actions in the region that favors the implementation of the male figure in this type of program.

## Figures and Tables

**Table 1:** Distribution of the variable Depression, according to sex.

Target Variable	Total Participants N (%)	Depressives N (%)	Non-Depressives N (%)
WOMEN	1901 (96.4)	685 (94.6)	1216 (97.4)
MEN	71 (3.6)	39 (5.4)	32 (2.6)

**Table 2:** Sociodemographic variables.

Target Variable	Depressives N (%)	Non-Depressives N (%)	*p* *
Age (years)	71.6 (7.1)	72.6 (6.8)	
Body Mass Index (kg/m^2^)	N (%)	N (%)	
<20	1 (0.1)	3 (0.2)	<0.01
20–25	87 (12)	177 (14.2)	
25–30	331 (45.7)	581 (46.6)	
>30	305 (42.1)	487 (39)	
Marital status	N (%)	N (%)	
Married	493 (68.1)	784 (62.8)	<0.01
Divorced	18 (2.5)	35 (2.8)	
Single	30 (4.1)	53 (4.2)	
Widowed	183 (25.3)	376 (30.1)	
Education	N (%)	N (%)	
No formal education	267 (36.9)	518 (41.5)	<0.01
Primary education	357 (49.3)	592 (47.4)	
Secondary education	68 (9.4)	96 (7.7)	
Superior studies	32 (4.4)	42 (3.4)	
Smoking	N (%)	N (%)	
+24 cigarettes/day	0	2 (0,2)	0.10
15–24 cigarettes/day	7 (1)	12 (1)	
5–14 cigarettes/day	14 (1.9)	26 (2.1)	
1–4 cigarettes/day	12 (1.7)	13 (1)	
No smoke	691 (95.4)	1195 (95.8)	
Alcohol consumption	N (%)	N (%)	
Every day one more than a glass	1 (0.1)	3 (0.2)	<0.01
2–3 times/week more than glass/day	7 (1)	18 (1.4)	
1 time/week more than 1 glass/day	11 (1.5)	37 (3)	
2–3 times/month more than 1 glass/day	23 (3.2)	18 (1.4)	
Less than 1 time/month more than 1 glass/day	21 (2.9)	30 (2.4)	
Never more than 1 glass/day	58 (8)	65 (5.2)	
Never drink alcohol	603 (83.3)	1089 (87.3)	
Physical activity (hour/week)	N (%)	N (%)	
0	24 (3.3)	53 (4.2)	<0.01
<3	35 (4.8)	40 (3.2)	
>3	665 (91.9)	1155 (92.5)	

* X2 Test.

**Table 3:** In relation to the Extremadura Health Service.

Target Variable	Depressives Mean (Sd)	Depressives Median (IQR)	Non-Depressives Mean (Sd)	Non-Depressives Mean(IQR)	*p* *
Falls	0.45 (1.32)	0 (1)	0.38 (1.19)	0 (0)	0.09
Primary Care Visits	3.92 (4.47)	3 (4)	2.41 (3.91)	2 (2)	<0.01
Visits_Nursing	3.92 (4.75)	2 (6)	2.80 (4.63)	1 (0)	<0.01
Specialist_Visits	1.38 (1.73)	1 (2)	1.31 (2.58)	1 (2)	0.01
Prescription_Visits	3.64 (3.96)	2 (5)	1.66 (3.02)	1 (1)	<0.01
Hospital_Days	0.19 (1.26)	0 (0)	0.31 (1.78)	0 (0)	0.20

Sd = standard deviation. IQR = interquartile range. * U de Mann Whitney. Table 4 shows the CFRS and HRQoL parameters. There are statistically significant differences between depressive and non-depressive patient in the variables of leg strength (<0.01), agility (<0.01), trunk flexibility (0.01), cardiorespiratory capacity (<0.01) and HRQoL (<0.01).

**Table 4:** Regarding physical condition and quality of life tests.

Target Variable	Depressives Mean (Sd)	Depressives Median (IQR)	Non-Depressives Mean (Sd)	Non-Depressives Mean (IQR)	*p* *
Handgrip	41.21 (9.84)	41.70 (12.8)	40.85 (9.61)	40.65 (12.2)	0.06
Seat-and-reach	0.41 (8,92)	2 (2.25)	1.87 (8.19)	1.6 (5.12)	0.01
Back scratch	−6.25 (8.36)	−5 (−11)	−6.31 (8.48)	−5 (−12)	0.97
Functional reach	25.49 (6.57)	25.20 (9)	25.78 (6.69)	26 (8)	0.16
Stand Up	12.81 (3.24)	13 (4)	14.71 (3.67)	14 (5)	<0.01
Time up and go	7.94 (1.88)	7.79 (2.03)	7.32 (2.00)	6.96 (1.9)	<0.01
6-min-walk	447.14 (110.06)	440 (120)	457.21 (86.72)	460 (110)	<0.01
EQ−5D utility index	0.81 (0.19)	0.87 (0.22)	0.91 (0.13)	1(0.12)	<0.01

Sd = standard deviation. IQR = interquartile range. * U de Mann Whitney.

## Data Availability

Data available upon request due to ethical and privacy restrictions. Data presented in this study are available upon request from the corresponding author.
